# Examining whether inflammation mediates effects of genetic risk and trauma on psychopathology

**DOI:** 10.1192/bjo.2025.10054

**Published:** 2025-08-15

**Authors:** Philippa Lilford, Gemma Hammerton, Golam M. Khandaker, Jeremy Hall, Stan Zammit, Hannah J. Jones

**Affiliations:** Centre for Academic Mental Health, Population Health Sciences, Bristol Medical School, University of Bristol, Bristol, UK; Avon and Wiltshire Mental Health Partnership NHS Trust, Bristol, UK; MRC Integrative Epidemiology Unit, Population Health Sciences, Bristol Medical School, University of Bristol, Bristol, UK; MRC Centre for Neuropsychiatric Genetics and Genomics, Division of Psychological Medicine and Clinical Neurosciences, Cardiff University School of Medicine, Cardiff University, Cardiff, UK; Hodge Centre for Neuropsychiatric Immunology, Cardiff University, Cardiff, UK; Neuroscience and Mental Health Innovation Institute, Cardiff University, Cardiff, UK; National Institute for Health Research Bristol Biomedical Research Centre, University Hospitals Bristol and Weston NHS Foundation Trust and University of Bristol, Bristol, UK

**Keywords:** Polygenic risk score, inflammation, psychopathology, IL-6, ALSPAC

## Abstract

**Background:**

Longitudinal studies have revealed that raised levels of inflammatory markers and trauma in childhood are associated with psychopathology in adulthood.

**Aims:**

To examine whether inflammation in childhood mediates the effects of genetic risk and trauma on psychopathology in early adulthood.

**Method:**

Measures of trauma exposure, inflammation and psychopathology were collected from the Avon Longitudinal Study of Parents and Children. Exposure to trauma was measured from 5 to 11 years of age; C-reactive protein and interleukin-6 levels were measured at 9 years; and depression, anxiety disorders, negative symptoms and psychotic experiences were assessed at 24 years. Polygenic risk scores (PRSs) were created for schizophrenia, depression, anxiety and psychotic experiences. Mediation analyses were conducted using imputed data (*N*: 7859 to 8700) to investigate whether inflammation mediated the associations of genetic risk and childhood trauma with psychopathology.

**Results:**

Most psychiatric PRSs were associated with multiple psychopathological outcomes in adulthood, with the exception of the PRS for psychotic experiences. Childhood trauma was associated with all psychopathology. However, there was no strong evidence that inflammatory markers in childhood mediated associations among PRSs, trauma and psychopathology. Sensitivity analyses using outcomes from age 18 and PRSs based on single-nucleotide polymorphisms that met more stringent standards of evidence of association gave results consistent with those of our primary analyses.

**Conclusions:**

We found little evidence that interleukin-6 or C-reactive protein mediated the pathway between genetic liability for psychiatric phenotypes or trauma and subsequent psychopathology. Longitudinal investigation of other inflammatory and non-inflammatory pathways is required to identify modifiable targets and inform novel treatment strategies for individuals at genetic or trauma-related risk of psychiatric illness.

Mental illnesses are common and disabling disorders with significant costs, comparable with those of physical illnesses, to both the individual and society. However, the aetiology of psychiatric illnesses is generally less well understood than that of physical illnesses, and psychiatric illnesses are diagnosed through classification systems which describe symptomatology rather than the underlying pathophysiology. Despite this, genome-wide association studies (GWAS) have provided evidence that psychiatric illnesses including schizophrenia and major depressive disorder have a strong genetic component,^1^ with twin studies estimating heritability to be 30–40% for depression^2^ and as high as 80% for schizophrenia.^3^ Although inheritance for these conditions is polygenic and influenced by numerous common genetic variants, pathway analysis of GWAS results has highlighted immune dysfunction as an important biological process underlying genetic vulnerability and therefore a potentially important causal mechanism to be investigated.^4^

Long before genetic associations were revealed, inflammation and immunity were proposed as potential mechanisms underlying mental illness owing to a number of epidemiological associations. For example, administration of lipopolysaccharide, a cell wall component of Gram-negative bacteria, produces a syndrome of depression like symptoms,^5^ and 25% of patients who receive treatment with interferon (a potent anti-viral immunomodulator) develop a major depressive episode within 48 weeks.^6^ In addition, a range of childhood infections are associated with an increased risk of schizophrenia in adulthood,^7^ and there is also some evidence that negative symptoms in schizophrenia are responsive to minocycline, a tetracyclic anti-inflammatory drug.^8^ There is also an increased prevalence of autoimmune disorder in people with psychiatric disorders compared with the general population.^9,10^ Moreover, having a family history of schizophrenia increases the risk of developing an autoimmune disorder, suggesting shared genetic factors between schizophrenia and autoimmune disorders.^9^ Finally, meta-analyses of cross-sectional and case–control studies have shown higher levels of circulating inflammatory biomarkers, namely interleukin-6 (IL-6) and C-reactive protein (CRP), in individuals with schizophrenia,^11^ depression^12^ and anxiety.^13^ However, confounding and reverse causation remain plausible alternative explanations for these associations. For example, associations between depression and inflammation may be confounded by factors such as obesity, or becoming depressed may lead to characteristics which increase inflammation, such as obesity and smoking. However, evidence from the population-based Avon Longitudinal Study of Parents and Children (ALSPAC) showed that higher IL-6 levels in childhood were associated with subclinical schizophrenia symptoms associated with psychotic disorders, namely psychotic experiences and negative symptoms, as well as depression in early adulthood, arguing against the latter and providing evidence of a possible causal pathway.^14^

In addition to genetic risk, trauma is an important risk factor to consider, as it is associated with both inflammation and psychopathology. Childhood trauma such as physical abuse and neglect is associated with a range of mental illnesses in adulthood, including anxiety disorders, mood disorders, personality disorders^15^ and psychotic disorders.^16^ Childhood trauma is also associated with raised inflammatory markers in the blood,^17^ with evidence that experiencing acute and chronic stress can lead to immune dysfunction in psychiatric illness through interplay with the hypothalamic–pituitary–adrenal axis.^18^ Therefore, chronic inflammation is a possible underlying mechanism of the association between trauma and psychopathology.

Despite evidence that genetic risk of psychiatric disorders and biological sequalae of childhood trauma both involve immune pathways, whether inflammation represents a mechanism linking genetic or key early life environmental risk factors with psychopathology in adulthood remains unclear. Here, using data from a longitudinal birth cohort, we examined whether inflammation was a potential mechanism underlying the associations between genetic liability to psychiatric disorder, childhood trauma and psychopathology (depression, psychotic experiences, negative symptoms and anxiety) in early adulthood. We investigated multiple outcomes and compared associations of raised inflammatory markers with a range of psychiatric phenotypes which may suggest potential common or unique underlying pathophysiology. We hypothesised that inflammation, measured using serum levels of IL-6 and CRP in individuals aged 9 years, would mediate the associations between (a) genetic liability for psychiatric disorders (quantified using polygenic risk scores (PRSs)) and psychopathology and (b) childhood trauma and psychopathology.

## Method

### Participants

The sample included participants from the Avon Longitudinal Study of Parents and Children (ALSPAC), a longitudinal cohort of 14 062 live births to pregnant women with expected dates of delivery between 1 April 1991 and 31 December 1992 in the Avon county in thesouth-west of England.^19^ The study website contains details of all the data that are available through a fully searchable data dictionary and variable search tool (http://www.bristol.ac.uk/alspac/researchers/our-data/). Study data were collected and managed using REDCap (Research Electronic Data Capture). REDCap is a secure, web-based software platform designed to support data capture for research studies hosted at the University of Bristol.

### Polygenic risk scores

Genetic data for 9912 ALSPAC participants were acquired using the Illumina HumanHap550 quad genome-wide single-nucleotide polymorphism (SNP) genotyping platform (see the Supplementary Material available at https://doi.org/10.1192/bjo.2025.10054 for further information). PRSs were created for each individual in ALSPAC for schizophrenia, anxiety, depression and psychotic experiences using effect sizes from publicly available large-scale GWAS summary statistics (see Supplementary Table 1 for discovery GWAS sample details) to weight SNP allele counts. PRSs were calculated using the PLINK (version 1.9) ‘score’ command (see the Supplementary Material for further information) and were standardised before use so that results are presented per standard deviation increase in PRS.

For the primary analysis, scores were constructed using SNPs with a GWAS training set *P*-value threshold of ≤0.5 and weighted using the logarithm of the odds ratio reported for each GWAS training set. To examine the consistency of the results when using PRSs based on SNPs with stronger evidence of psychiatric phenotype association, analyses were repeated, and the results were compared using PRSs generated using SNPs meeting GWAS training set *P*-value thresholds of ≤0.05 and ≤5 × 10^−8^.

### Inflammatory markers

Measures of CRP and IL-6 were taken from non-fasting children at age 9 years. Blood samples were analysed in 2008 after a median of 7.5 years in storage. High-sensitivity CRP was measured by automated particle-enhanced immunoturbidimetric assay (Roche) and IL-6 by enzyme-linked immunosorbent assay (R&D Systems). Interassay coefficients of variation were less than 5%. In the total sample, IL-6 values ranged from 0.007 to 20.05 pg/mL (*n* = 5071), and CRP values ranged from 0.01 to 67.44 mg/L (*n* = 5081). All individuals with CRP and IL-6 data at age 9 were included in our primary analyses. Sensitivity analyses were also conducted following removal of individuals who showed indication of an active disease process (CRP level >10 mg/L) or reported an infection at the time of blood collection or in the preceding week.

### Psychopathology outcomes

Outcomes of interest included depression, anxiety disorders, psychotic experiences and negative symptoms. Outcomes at age 24 years were examined in the primary analysis, with outcomes at (or near) age 18 years used in sensitivity analyses to investigate the robustness of associations. Primary outcomes at age 24 were chosen as more cases of psychiatric disorders have emerged by early adulthood.

#### Depression

Depression was assessed through the self-administered Clinical Interview Schedule-Revised at ages 18 and 24 years.^20^ This measure provides diagnoses of mild, moderate or severe depression based on ICD-10 criteria over the past month. As our primary depression measure, we examined a binary outcome of moderate and severe depression versus mild or none.

#### Anxiety

Anxiety was also assessed through the Clinical Interview Schedule-Revised at ages 18 and 24 years. The following anxiety disorders were included: generalised anxiety disorder, social phobia, specific phobia and panic disorder. The decision was made to consider a spectrum of anxiety disorders given their core phenotypic features and shared genetic risk.^21^ A single binary variable determining the presence or not of any anxiety disorder was then created.

#### Psychotic experiences

Psychotic experiences were assessed through semi-structured interviews using the Psychosis-Like Symptom Interview, which has a kappa score of 0.8, indicating good interrater reliability,^22^ and is used to assess the presence of 12 core psychotic experiences including hallucinations, delusions and thought interference. Participants who answered ‘maybe’ or ‘yes’ to stem questions were then cross-questioned by interviewers and coded according to the Schedules for Clinical Assessment in Neuropsychiatry. Interviewers rated experiences as ‘not present’, ‘suspected’ or ‘definite’. We examined a binary outcome of having at least one definite psychotic experience (compared with suspected or absent psychotic experiences) since age 12 (at age 24 years) or in the past 6 months (at age 18 years).

#### Negative symptoms

Negative symptoms were assessed using ten negative-symptom-related questions from the Community Assessment of Psychic Experiences, a self-report questionnaire which has been well validated with respect to its ability to detect negative symptoms.^23^ The questions measure past year (at age 24 years) or past month (at age 16 years) negative symptoms such as anergia, apathy and asociality and the frequency of their occurrence (0, never; 1, sometimes; 2, often; 3, always). A total score was created from the sum of the responses (minimum score 0, maximum score 30), and as a primary measure we created a binary variable using the top decile as a cut-off.

#### Trauma

A binary variable indexing the presence or absence of trauma (including exposure to physical, sexual and emotional abuse, emotional neglect, domestic violence and bullying) between the ages of 5 and 11 years was derived on the basis of responses by parents and participants to 121 questions about traumatic events (see Croft et al^24^ for more detail). All data on presence or absence of trauma were collected contemporaneously, with the exception of reports of sexual abuse and self-reports of emotional neglect and physical abuse, which were supplemented using a retrospective questionnaire completed at age 22 years.

### Confounders

**Fig. 1:**
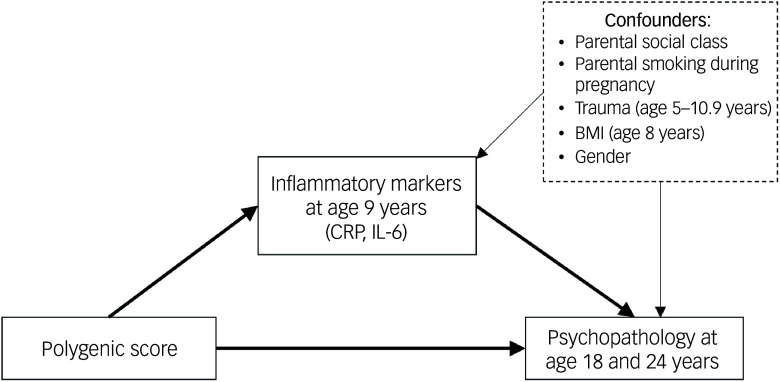
Modelling of the mediating effects of inflammation on associations between genetic liability to psychiatric disorders and psychopathology in adulthood. BMI, body mass index; CRP, C-reactive protein; IL-6, interleukin-6.

**Fig. 2:**
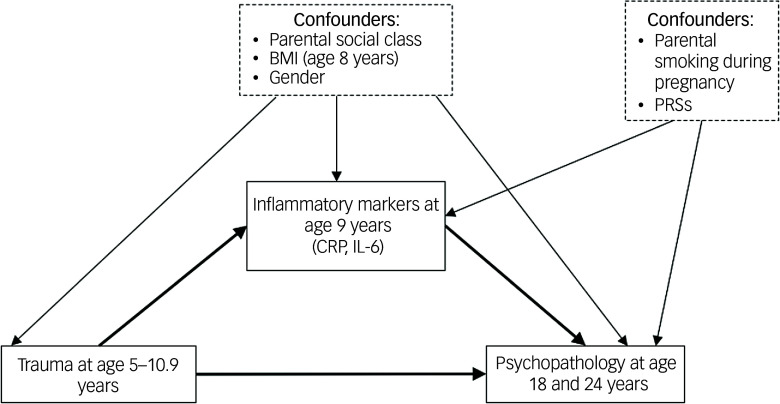
Modelling of the mediating effects of inflammation on the associations between trauma and psychopathology in adulthood. BMI, body mass index; CRP, C-reactive protein; IL-6, interleukin-6; PRSs, polygenic risk scores.

Potential confounders of the inflammation–psychopathology pathway included: maternal and paternal smoking during pregnancy (binary measures), body mass index (BMI) at age 8 years, gender at birth, parental social class (highest of either parent) and either history of trauma (when PRS was the exposure) or PRS (when trauma was the exposure). Parental social class, BMI and gender at birth were also included as potential confounders of the trauma–psychopathology pathway (see Figs. 1 and 2 for details of confounders adjusted for in each pathway). Analyses examining PRSs as the exposures were further adjusted for the first ten genetic principal components to account for population stratification. Comparisons between individuals experiencing and not experiencing each psychopathology symptom for each covariate are shown in Supplementary Tables 2–5.

### Missing data

Within our largest analysis sample (individuals with data on presence or absence of trauma from 5 to 11 years; *n* = 8700), data missingness ranged from approximately 62 to 63% for our outcomes (depression, anxiety disorder, negative symptoms and psychotic experiences at age 24) and was approximately 50% for our mediators (CRP and IL-6 at age 9) (Table 1). As such, we performed multivariate imputation by chained equations^25^ with 50 imputations, up to the sample with exposure data (PRS analyses: *n* = 7859; trauma analyses: *n* = 8700). To make the missing at random assumption more plausible, together with all analysis variables, we included auxiliary variables that were strongly correlated with the observed values of the exposure or covariates and had the potential to also be predictive of missingness on those variables within our imputation models. These were earlier measurements of the outcomes from ages 18 and 16 years, a family history of mental health problems, an earlier measurement of BMI from age 7 years, mother’s highest educational qualification and home ownership status, a later measure of CRP from age 16 years and a genetic variant strongly related to circulating soluble IL-6 receptor and IL-6 concentrations (rs2228145).^26^ Details of the variables within our imputation models are presented in Supplementary Table 6. All results of regression and mediation analyses were pooled across imputed data-sets using Rubin’s rules.^25^

**Table 1:** Baseline characteristics of participants before and after imputation and proportion of missingness

	Original		Imputed^a^
Continuous variables	Mean (s.d.)	Missingness (%)^b^	Mean range
CRP age 9 years	0.79 (2.74)	4336/8700 (49.84)	0.75–0.87
IL-6 age 9 years	1.27 (1.53)	4346/8700 (49.95)	1.25–1.31
BMI age 8 years	17.13 (2.39)	2808/8700 (32.28)	17.14–17.18
Categorical variables	Frequency (%)	Missingness (%)^b^	Frequency range (%)
Psychotic experiences (24 years)	321/3262 (9.84)	5438/8700 (62.51)	10.03–11.97
Negative symptoms (24 years)	291/3207 (9.07)	5493/8700 (63.14)	9.66–11.77
Depression (24 years)	244/3322 (7.34)	5378/8700 (61.82)	7.03–8.64
Anxiety disorder (24 years)	326/3300 (9.88)	5400/8700 (62.07)	8.94–10.98
Gender at birth (female)	4276/8685 (49.23)	15/8700 (0.17)	49.18–49.28
Mother smoked in pregnancy	1945/8477 (22.94)	223/8700 (2.56)	22.92–23.30
Father smoked in pregnancy	3108/6712 (46.31)	1988/8700 (22.85)	45.01–46.43
Parental social class			
Professional	297/7999 (3.71)	701/8700 (8.06)	3.53–3.74
Managerial	2030/7999 (25.38)		24.56–25.07
Skilled (non-manual)	2115/7999 (26.44)		25.85–26.43
Skilled (manual)	2163/7999 (27.04)		26.97–27.52
Partly skilled	1094/7999 (13.68)		13.93–14.43
Unskilled	300/7999 (3.75)		3.82–4.15
Trauma 5–11 years	3663/8700 (42.10)	–	–

CRP, C-reactive protein; IL-6, interleukin-6; BMI, body mass index.

a Ranges based on 50 imputed data-sets.

b Number of missing observations/largest analysis sample (percentage missing).

### Statistical analysis

Univariable and multivariable logistic and linear regressions were first used to estimate the total effects of PRSs and childhood trauma on psychiatric outcomes at 24 years, as well as associations between PRSs, childhood trauma and inflammatory markers (exposure–mediator associations) and between inflammatory markers and psychiatric outcomes at 24 years (mediator–outcome associations).

Mediation models were then used to assess (a) the indirect effects of psychiatric phenotype PRSs on psychopathology via inflammatory markers and (b) the indirect effects of trauma on psychopathology via inflammatory markers. Mediation analysis was undertaken for associations between each PRS and its most closely related psychopathology and/or where there was strong evidence of a total effect. Mediation models were run for IL-6 and CRP separately using generalised structural equation modelling, and standard errors for indirect effects were calculated using bootstrapping with 1000 replications. Direct and indirect effects were obtained using the product of coefficients approach, whereby the indirect effects are derived by multiplying the parameters along the paths from exposure to mediator and mediator to outcome. This approach is appropriate for use with a binary outcome provided the outcomes are rare (10% is generally used as a cut-off).^27^

On the basis of the number of total effects we estimated for each analysis, we conducted conservative Bonferroni corrections and considered there to be evidence of association when *P* ≤ 0.003 for analyses where PRSs were exposures (four PRSs per four outcomes 0.05/16) and *P* ≤ 0.0125 for analyses where trauma was the exposure (four outcomes, 0.05/4).

All analyses were conducted using STATA version 17.0 for macOS. Scripts for imputation and mediation analyses are available from https://anonymous.4open.science/r/BJPsychOpen_20231212.

### Ethics statement and consent

Ethics approval for ALSPAC clinic data collection was obtained from the ALSPAC Ethics and Law Committee and the local research ethics committees. More details of the specific approval references for the main study and each subsequent clinic can be found at https://www.bristol.ac.uk/media-library/sites/alspac/documents/governance/Research_Ethics_Committee_approval_references.pdf. All self-completion questionnaire content was approved by the ALSPAC Ethics and Law Committee.

Informed written consent for the use of data collected via clinics was obtained from participants following the recommendations of the ALSPAC Ethics and Law Committee at the time. Consent for biological samples was collected in accordance with the Human Tissue Act (2004). Consent was implied by the participant returning a completed postal questionnaire or submitting an online questionnaire.

## Results

Baseline characteristics of analysis sample participants before and after imputation and the proportion of missingness are shown in Table 1.

### Mediating effects of raised levels of inflammatory markers at age 9 years on associations of genetic risk and childhood trauma with adult psychopathology at age 24 years

We observed strong evidence of a total effect between the schizophrenia PRS and psychotic experiences (odds ratio per standard deviation increase in PRS 1.22, 95% CI 1.08 to 1.37, *P* = 0.001); between the depression PRS and negative symptoms (odds ratio per standard deviation increase in PRS 1.34, 95% CI 1.18 to 1.51, *P* < 0.001), depression (odds ratio per standard deviation increase in PRS 1.41, 95% CI 1.23 to 1.62, *P* < 0.001) and anxiety disorder (odds ratio per standard deviation increase in PRS 1.22, 95% CI 1.07 to 1.39, *P* = 0.003); and between the anxiety PRS and negative symptoms (odds ratio per standard deviation increase in PRS 1.30, 95% CI 1.17 to 1.45, *P* < 0.001), depression (odds ratio per standard deviation increase in PRS 1.26, 95% CI 1.12 to 1.43, *P* < 0.001) and anxiety disorder (odds ratio per standard deviation increase in PRS 1.19, 95% CI 1.07 to 1.32, *P* = 0.002) (Supplementary Table 7). We also observed strong evidence of a total effect between experiencing trauma between ages 5 and 11 and all outcomes (psychotic experiences: adjusted odds ratio 1.87, 95% CI 1.47 to 2.37, *P* < 0.001; negative symptoms: adjusted odds ratio 2.16, 95% CI 1.69 to 2.77, *P* < 0.001; depression: adjusted odds ratio 2.31, 95% CI 1.79 to 2.97, *P* < 0.001; anxiety disorder: adjusted odds ratio 1.69, 95% CI 1.36 to 2.09, *P* < 0.001) (Supplementary Table 8).

There was no strong evidence of associations between PRSs, childhood trauma and IL-6 or CRP at age 9 (Supplementary Table 9) or between IL-6 or CRP at age 9 and psychiatric outcomes at 24 years (Supplementary Table 10), except for IL-6 and negative symptoms at age 24 years (odds ratio 1.14 per unit increase in IL-6, 95% CI 1.05 to 1.23, *P* = 0.001). We also found no strong evidence of mediation between PRSs for various psychiatric phenotypes and psychopathology at age 24 years through IL-6 or CRP (Table 2), with direct effects between PRSs and psychiatric phenotypes being almost identical to the total effect. Similarly, mediation analysis revealed little evidence of mediation between trauma and psychiatric outcomes through IL-6 or CRP (Table 3).

**Table 2:** Mediation analysis results for the association between PRSs for psychiatric phenotypes and psychopathology at age 24 through IL-6 and CRP at age 9 (*n* = 7859)^a^

PRS	Outcome	Total effect Odds ratio (95% CI)	Direct effect Odds ratio (95% CI)	Indirect effect Odds ratio (95% CI)
Mediation through IL-6				
Psychotic experiences	Psychotic experiences	1.06 (0.94 to 1.20)	1.06 (0.94 to 1.20)	1.00 (1.00 to 1.00)
Schizophrenia	Psychotic experiences	1.20 (1.07 to 1.34)	1.20 (1.07 to 1.34)	1.00 (1.00 to 1.00)
Depression	Negative symptoms	1.27 (1.12 to 1.45)	1.27 (1.11 to 1.44)	1.00 (1.00 to 1.01)
Depression	Depression	1.34 (1.16 to 1.55)	1.34 (1.16 to 1.55)	1.00 (1.00 to 1.01)
Depression	Any anxiety disorder	1.17 (1.02 to 1.34)	1.17 (1.02 to 1.34)	1.00 (1.00 to 1.01)
Anxiety	Negative symptoms	1.27 (1.14 to 1.43)	1.27 (1.14 to 1.43)	1.00 (0.99 to 1.01)
Anxiety	Depression	1.24 (1.09 to 1.40)	1.24 (1.09 to 1.40)	1.00 (1.00 to 1.00)
Anxiety	Any anxiety disorder	1.16 (1.05 to 1.30)	1.17 (1.05 to 1.30)	1.00 (1.00 to 1.00)
Mediation through CRP				
Psychotic experiences	Psychotic experiences	1.06 (0.94 to 1.20)	1.06 (0.94 to 1.20)	1.00 (1.00 to 1.00)
Schizophrenia	Psychotic experiences	1.20 (1.07 to 1.35)	1.20 (1.06 to 1.35)	1.00 (1.00 to 1.00)
Depression	Negative symptoms	1.27 (1.12 to 1.45)	1.27 (1.12 to 1.45)	1.00 (1.00 to 1.00)
Depression	Depression	1.34 (1.16 to 1.55)	1.34 (1.16 to 1.55)	1.00 (1.00 to 1.00)
Depression	Any anxiety disorder	1.17 (1.02 to 1.34)	1.17 (1.02 to 1.34)	1.00 (1.00 to 1.00)
Anxiety	Negative symptoms	1.27 (1.14 to 1.42)	1.27 (1.14 to 1.42)	1.00 (1.00 to 1.00)
Anxiety	Depression	1.23 (1.09 to 1.40)	1.23 (1.09 to 1.39)	1.00 (1.00 to 1.00)
Anxiety	Any anxiety disorder	1.16 (1.04 to 1.30)	1.16 (1.04 to 1.30)	1.00 (1.00 to 1.00)

PRS, polygenic risk score; IL-6, interleukin-6; CRP, C-reactive protein.

a. All analyses were adjusted for trauma (age 5–11 years), parental smoking, parental social class, gender and body mass index (age 8).

**Table 3:** Mediation analysis results for the association between trauma (age 5–10) and psychopathology at age 24 through IL-6 and CRP at age 9 (*n* = 8700)^a^

Outcome	Total effect Odds ratio (95% CI)	Direct effect Odds ratio (95% CI)	Indirect effect via mediator Odds ratio (95% CI)
Mediation through IL-6			
Psychotic experiences	1.73 (1.35 to 2.22)	1.73 (1.35 to 2.21)	1.00 (1.00 to 1.01)
Negative symptoms	1.96 (1.52 to 2.53)	1.95 (1.51 to 2.52)	1.01 (0.99 to 1.02)
Depression	2.07 (1.60 to 2.68)	2.06 (1.60 to 2.67)	1.00 (0.99 to 1.01)
Any anxiety disorder	1.57 (1.27 to 1.94)	1.56 (1.26 to 1.93)	1.00 (1.00 to 1.01)
Mediation through CRP			
Psychotic experiences	1.73 (1.35 to 2.22)	1.73 (1.35 to 2.22)	1.00 (0.99 to 1.01)
Negative symptoms	1.96 (1.52 to 2.53)	1.95 (1.51 to 2.51)	1.00 (1.00 to 1.01)
Depression	2.07 (1.60 to 2.68)	2.06 (1.60 to 2.67)	1.00 (1.00 to 1.01)
Any anxiety disorder	1.57 (1.27 to 1.94)	1.57 (1.27 to 1.94)	1.00 (1.00 to 1.01)

IL-6, interleukin-6; CRP, C-reactive protein.

a. All analyses were adjusted for parental smoking, parental social class, gender and body mass index (age 8) and polygenic scores for psychotic experiences, schizophrenia, depression and anxiety.

### Sensitivity analyses

Consistent with our primary analyses, there was little evidence that IL-6 or CRP mediated the associations between PRSs (Supplementary Table 11) or trauma (Supplementary Table 12) and psychopathology at age 18 years (or near). There was also no strong evidence that IL-6 or CRP mediated the associations between PRSs and psychopathology at age 24 years when analyses were repeated using SNPs meeting GWAS training set *P*-value thresholds of ≤0.05 and ≤5 × 10^–8^ (Supplementary Tables 13 and 14). Results were consistent between analyses using the imputed sample and complete case sample (Supplementary Tables 15 and 16) and the sample excluding individuals with CRP >10 mg/L or reporting infection at the time of blood collection (Supplementary Tables 17 and 18).

## Discussion

To our knowledge, this is the first study to examine whether raised inflammatory markers in childhood mediate the effects of genetic risk and risk from trauma on psychopathology in early adulthood. Our study, which used a large, well-characterised prospective birth cohort with strategies implemented to address missing data, confirmed previous research which demonstrated that childhood trauma and PRSs for different psychiatric disorders confer risk of a range of psychopathologies in adulthood.^15,28^ However, although immune dysfunction has been implicated as a possible biological pathway in psychopathology, we found little evidence that raised IL-6 or CRP levels in childhood mediated these associations.

Previous longitudinal studies have demonstrated association of raised IL-6 but not CRP levels in childhood with depression, depressive episodes, psychotic experiences and negative symptoms in late adolescence and/or early adulthood.^14,29^ Mendelian randomisation analyses, which provide evidence of causality if certain assumptions are met, have also indicated that IL-6 may lie on the causal path to development of depression and psychosis,^30^ whereas higher CRP levels are protective^31^ – although the latter finding may be explained by a potential differential role of IL-6 classic and *trans* signalling on illness development.^30^

Taken at face value, our results indicate that although inflammation in childhood may influence psychopathology, it is not a downstream consequence of trauma or genetic risk of psychiatric phenotypes. One explanation is that although immune dysfunction has been considered as an important mechanism underlying genetic vulnerability to psychiatric disorders,^4^ and childhood trauma is also associated with raised inflammatory markers,^17^ dysregulated inflammation as a consequence of these risk factors may emerge later than childhood. For example, we may find stronger evidence of mediation when investigating inflammation at a time when individuals are more likely to start risk-taking behaviours that lead to an increase in inflammation. In fact, childhood trauma has been shown to have modest to strong associations with risky behaviours such as alcohol and drug use,^32^ and genetic studies have shown a polygenic overlap between risk-taking and a range of psychiatric disorders.^33^ This explanation is supported by a study showing that adverse childhood experiences are associated with composite inflammatory marker glycoprotein acetyls later in life but not during adolescence; however, this study also found little evidence of association at ages 18 or 24 years, when some risk-taking behaviours may have already been adopted.^34^

Another explanation, and potential limitation, is that our measurement of inflammation, which was a one-off measurement of IL-6 and CRP levels in childhood, may not have captured the chronic inflammation thought to be important in the pathophysiology of psychiatric disorders.^14^ A recent systematic review found that although one-off measures are adequate for index inflammation up to 6 months, the stability of these measures is generally low over 5 years.^35^ Such long-term variability may be due to transient rises in inflammatory markers caused by acute infection, among other causes, which may have different associations with subsequent risk of developing psychiatric phenotypes than chronic low-grade inflammation. Having serial measurements of IL-6 and CRP over time that are more temporally associated with the outcomes of interest would allow more robust examination of whether inflammation is associated with future risk of psychopathology and whether acute or chronic infection is likely to be more important. For example, using three measures of CRP from ages 9 to 18 years, Osimo et al^36^ showed that individuals who were characterised as having increasing CRP levels over time had a higher risk of moderate or severe depression at age 18 years. Repeated measures could also enable testing of critical or sensitive period effects, whereby the risk of psychopathology may be different if the brain is exposed to inflammation during different stages of development. A study from Sweden, for example, showed that lower levels of inflammatory markers in neonates were associated with an increased risk of schizophrenia in adulthood,^37^ which was not consistent with findings that raised IL-6 levels in childhood are associated with an increased risk of psychosis in early adulthood.^14^

Furthermore, the highest value of CRP levels in this study was 67.44 mg/L, and so our sample included individuals with an indicated active disease process (CRP > 10 mg/L), although the significance of this threshold has recently been disputed.^38^ As such, our mediator may have captured both chronic and acute inflammation. We did not exclude individuals with high levels of CRP or IL-6 from our main analyses, as it was unknown whether these levels represented acute illness or chronic inflammation or were due to other factors (e.g. a heritable propensity for high CRP).^38^ Nevertheless, in our conservative sensitivity analyses following exclusion of individuals with high CRP levels or a reported infection, we found that results were consistent with our main analyses.

Another limitation of the current study was that the trauma variable used in this study was measured at ages 5–11 years, whereas inflammatory markers were measured at age 9. This slight overlap in age between the exposure and mediator meant that for some individuals, the temporal relationship needed for robust inference from mediation analyses was not met. However, as we found little evidence of mediation in our study, the overlap in age did not result in any false positive results.

Furthermore, although inflammation has been shown to be associated with childhood trauma and has been implicated as a potential risk factor in psychiatric conditions, effect sizes tend to be small.^17,39^ As such, our mediation analyses may not have been sufficiently powered to estimate indirect effects with good precision. *Post hoc* power calculations suggested that our analyses were well powered to estimate indirect effects through inflammation between trauma and psychopathology (99% power); however, some of our analyses estimating indirect effects through inflammation between PRSs and psychopathology may have been underpowered (power range: 6–94%; see Supplementary Methods for details).

Attrition is an established challenge in longitudinal epidemiological research and may affect the generalisability of findings.^40^ Participation in the ALSPAC cohort has gradually declined over time, with lower participation associated with factors suggesting greater social disadvantage.^41^ This decrease in participation was evidenced in our complete case sample, where levels of missing data were higher at age 24 years than, for example, at age 9 years. We aimed to address the missingness in our analysis samples using multiple imputation, which assumes that data are missing at random given the variables included in the imputation model. Although the results were consistent between analyses using the imputed sample and complete case sample, we acknowledge that assumptions regarding the missing data mechanisms are untestable and that results can still be biased if the imputation model is mis-specified.

A final limitation of this study was that we did not account for heterogeneity within our outcomes. Depression, for example, is a heterogenous condition, and it may be that there is a subgroup of patients who develop MDD as a result of chronic inflammation. A systematic review found that a quarter of depressed patients exhibited low-grade inflammation, and over half of the patients had raised CRP levels.^12^ Similarly, schizophrenia is a complex syndrome with a wide variety of phenotypes. One study found that using molecular profiles, patients with schizophrenia could be separated into two subgroups: one with abnormalities predominantly in the immune molecules, and the other with abnormalities in growth factors and hormones.^42^ Such heterogeneity may have reduced power in our analyses; however, it is worth noting that we were still able to observe evidence of a direct effect from our genetic risk scores and trauma to our outcomes of interest.

## Supporting information

Lilford et al. supplementary material Lilford et al. supplementary material

## Data Availability

Data are available to bona fide researchers on application to the Avon Longitudinal Study of Parents and Children (ALSPAC) committee (https://www.bristol.ac.uk/alspac/researchers/access/).

## Supplementary material

The supplementary material is available online at https://doi.org/10.1192/bjo.2025.10054
