# Supplementary material for: Examining whether inflammation mediates effects of genetic risk and trauma on psychopathology
Source: BJPsych Open. 2025 Aug 15;11(5):e181. doi: 10.1192/bjo.2025.10054 (PMC12451734; doi:10.1192/bjo.2025.10054)
Supplement: Lilford et al. supplementary material [file S2056472425100549sup001.docx]

**The mediating effect of inflammatory markers on the association between genetic risk for psychiatric disorders and psychopathological outcomes – Supplementary Material**

## Supplementary Methods

**Genetic data**

Avon Longitudinal Study of Parents and Children (ALSPAC) participant genetic data were acquired using the Illumina HumanHap550 quad genome-wide single nucleotide polymorphism (SNP) genotyping platform from 9912 participants.

Individuals were excluded from further analysis on the basis of gender mismatches, minimal or excessive heterozygosity, disproportionate levels of individual missingness (>1%), evidence of cryptic relatedness (>10% of alleles identical by descent) and being of non-European ancestry (assessed by multidimensional scaling analysis including HapMap 2 individuals). SNPs with a minor allele frequency (MAF) of < 1%, Impute2 information quality metric of < 0.8, a call rate of < 95% or evidence for violations of Hardy-Weinberg equilibrium (p value < 5 x 10^-7^) were removed. Imputation of the target data was performed following haplotype estimation using ShapeIT (v2.r644) which utilises relatedness during phasing. Imputation was performed using Impute V2.2.2 against the 1000 genomes reference panel (Phase 1, Version 3; all polymorphic SNPs excluding singletons), using 2,186 reference haplotypes (including non-Europeans). Following quality control assessment and imputation, restricting to 1 young person per family, and excluding individuals who had withdrawn consent, genetic data was available for 477,482 SNPs for 7,977 ALSPAC participants.

**Polygenic scores**

Polygenic scores for schizophrenia, anxiety, depression and psychotic experiences were constructed for each ALSPAC individual using the PLINK (v1.9) ‘score’ command.^1 2^ For each score, SNP allele counts were weighted using SNP effect sizes from the most up to date, large scale genome-wide association studies (GWASs). Details of the GWASs can be reviewed in Table S1 below. Prior to construction of scores, SNPs were removed from the analysis if they had a minor allele frequency less than 0.01, an imputation quality less than 0.8 or if there was allelic mismatch between samples. Due to the high linkage disequilibrium (LD) within the extended major histocompatibility complex (MHC; chromosome 6: 25-34Mb) only a single SNP was included to represent this region. SNPs were pruned for LD using the PLINK ‘clump’ command to remove SNPs in LD (r^2^ > 0.25) with a more significant SNP in the training set. Windows of 500kb were used to assess inter-SNP LD for pruning. All polygenic scores were standardised (mean or 0, standard deviation or 1) before use.

**Post hoc power calculations**

Power calculations for mediation analyses were performed using a Monte Carlo simulation approach^3^ through the Monte Carlo Power Analysis for Indirect Effects web application (<https://schoemanna.shinyapps.io/mc_power_med/>, 5000 replications, 20,000 draws/replication).

For analyses of the mediating effect of inflammation on the association between trauma and psychopathology, assuming the following standardised effect sizes: i) trauma to psychopathology: 0.51,^4^ ii) trauma to inflammation: 0.08,^5^ and iii) inflammation to psychopathology: 0.04,^6^ our sample size of 8700 had 99% power to estimate an indirect effect with a 95% confidence level.

For analyses of the mediating effect of inflammation on the association between PRSs and psychopathology, assuming the following standardised effect sizes: i) PRS to psychopathology: 0.08 (calculated using the association between depression PRS and moods and feelings depression score at age 16 years in ALSPAC), and ii) inflammation to depression: 0.04,^6^ our sample size of 7859 had 6% to 94% power to estimate an indirect effect with a 95% confidence level, assuming a PRS to inflammation standardised effect size of 0.004 and 0.05, respectively. These effect sizes were taken from the lowest (anxiety PRS to IL-6) and highest (psychotic experiences PRS to CRP) absolute PRS to inflammation effect sizes reported in Table S9.

**Supplementary Material References**

1. Purcell S, Neale B, Todd-Brown K, et al. PLINK: A tool set for whole-genome association and population-based linkage analyses. *The American Journal of Human Genetics* 2007;81(3):559-75. doi: 10.1086/519795

2. Chang C, Chow C, Tellier L, et al. Second-generation PLINK: rising to the challenge of larger and richer datasets. *GigaScience* 2015;4(7)

3. Schoemann AM, Boulton AJ, Short SD. Determining power and sample size for simple and complex mediation models. *Social Psychological and Personality Science* 2017;8(4):379-86. doi: 10.1177/1948550617715068

4. Vibhakar V, Allen LR, Gee B, Meiser-Stedman R. A systematic review and meta-analysis on the prevalence of depression in children and adolescents after exposure to trauma. *Journal of Affective Disorders* 2019;255:77-89. doi: 10.1016/j.jad.2019.05.005 [published Online First: 20190506]

5. Baumeister D, Akhtar R, Ciufolini S, et al. Childhood trauma and adulthood inflammation: a meta-analysis of peripheral C-reactive protein, interleukin-6 and tumour necrosis factor-alpha. *Molecular Psychiatry* 2016;21(5):642-9. doi: 10.1038/mp.2015.67 [published Online First: 20150602]

6. Mac Giollabhui N, Ng TH, Ellman LM, Alloy LB. The longitudinal associations of inflammatory biomarkers and depression revisited: systematic review, meta-analysis, and meta-regression. *Molecular Psychiatry* 2021;26(7):3302-14. doi: 10.1038/s41380-020-00867-4 [published Online First: 20200817]

7. Legge SE, Jones HJ, Kendall KM, et al. Association of Genetic Liability to Psychotic Experiences with Neuropsychotic Disorders and Traits. *JAMA Psychiatry* 2019 doi: 10.1001/jamapsychiatry.2019.2508

8. Pardiñas AF, Holmans P, Pocklington AJ, et al. Common schizophrenia alleles are enriched in mutation-intolerant genes and in regions under strong background selection. *Nature Genetics* 2018;50(3):381-89. doi: 10.1038/s41588-018-0059-2

9. Howard DM, Adams MJ, Clarke TK, et al. Genome-wide meta-analysis of depression identifies 102 independent variants and highlights the importance of the prefrontal brain regions. *Nature Neuroscience* 2019;22(3):343-52. doi: 10.1038/s41593-018-0326-7

10. Purves KL, Coleman JRI, Meier SM, et al. A major role for common genetic variation in anxiety disorders. *Molecular Psychiatry* 2019:1-12. doi: 10.1038/s41380-019-0559-1

**Supplementary Tables**

Table S1: Details of genome wide association studies used to inform polygenic risk score single nucleotide polymorphism weightings

| **Phenotype** | **Author** | **Sample** | **Ancestry** | **Sample size** | **Cases** | **Controls** | **SNP *h^2^*** |
| --- | --- | --- | --- | --- | --- | --- | --- |
| Psychotic experiences | Legge et al. 2019^7^ | UK Biobank | European | 127,966 | 6,123 | 121,843 | 0.02^b^ |
| Schizophrenia | Pardiñas et al. 2018^8^ | CLOZUK and PGC | 97% European, 3% Asian | 105,318 | 40,675 | 64,643 | 0.23^c^ |
| Depression | Howard et al. 2019^9^ | UK Biobank and PGC^a^ | European | 500,199 | 170,756 | 329,443 | 0.09^d^ |
| Lifetime Anxiety Disorder | Purves et al. 2019^10^ | UK Biobank | Western European | 83566 | 25,453 | 58,113 | 0.26^e^ |

^a^ not including 23andMe

^b^ on observed scale

^c^ on liability scale, assuming schizophrenia prevalence of 0.007

^d^ on liability scale, assuming depression prevalence of 0.302

^e^ on liability scale, assuming anxiety disorder prevalence of 0.200

Table S2. Comparison for each covariate between individuals with and without psychotic experiences at age 24 years in the observed, non-imputed data

|  |  |  | Psychotic experiences at 24y | |  |
| --- | --- | --- | --- | --- | --- |
| Measure | |  | Yes | No | ***P*** |
| Psychotic experiences PRS | | N | 222 | 2197 | 0.30 |
|  |  | Mean | 0.10 | 0.004 |  |
|  |  | SD | 1.00 | 0.98 |  |
| Schizophrenia PRS | | N | 222 | 2197 | 0.004 |
|  |  | Mean | 0.07 | -0.10 |  |
|  |  | SD | 1.00 | 1.01 |  |
| Depression PRS | | N | 222 | 2197 | 0.06 |
|  |  | Mean | -0.001 | -0.1 |  |
|  |  | SD | 1.02 | 1.01 |  |
| Anxiety PRS | | N | 222 | 2197 | 0.15 |
|  |  | Mean | 0.05 | -0.04 |  |
|  |  | SD | 1.08 | 1.01 |  |
| CRP age 9 | | N | 184 | 1856 | 0.87 |
|  |  | Mean | 0.77 | 0.77 |  |
|  |  | SD | 1.70 | 2.70 |  |
| IL-6 age 9 | | N | 184 | 1853 | 0.32 |
|  |  | Mean | 1.40 | 1.27 |  |
|  |  | SD | 1.49 | 1.54 |  |
| BMI age 8 | | N | 244 | 2377 | <0.001 |
|  |  | Mean | 17.65 | 17.07 |  |
|  |  | SD | 2.86 | 2.32 |  |
| Sex | Male | N (%) | 117 (9.41) | 1127 (90.59) | 0.51 |
|  | Female | N (%) | 204 (10.12) | 1812 (89.88) |  |
| Mother smoked in pregnancy | No | N (%) | 237 (8.83) | 2446 (91.17) | <0.001 |
|  | Yes | N (%) | 79 (15.05) | 446 (84.95) |  |
| Father smoked in pregnancy | No | N (%) | 130 (8.13) | 1469 (91.87) | <0.001 |
|  | Yes | N (%) | 127 (13.01) | 849 (86.99) |  |
| Parental social class | Professional | N (%) | 12 (7.36) | 151 (92.64) | 0.17 |
|  | Managerial | N (%) | 76 (8.37) | 832 (91.63) |  |
|  | Skilled (non-manual) | N (%) | 86 (9.82) | 790 (90.18) |  |
|  | Skilled (manual) | N (%) | 78 (10.89) | 638 (89.11) |  |
|  | Partly skilled | N (%) | 44 (12.75) | 301 (87.25) |  |
|  | Unskilled | N (%) | 9 (11.25) | 71 (88.75) |  |
| Trauma 5-11 years | No | N (%) | 146 (7.64) | 1766 (92.36) | <0.001 |
|  | Yes | N (%) | 175 (12.96) | 1175 (87.04) |  |

**Note:** PRS, polygenic risk scores; SD, standard deviation; BMI, body mass index; *P*, *P* value for t-test (continuous measures) or chi-squared test (categorical measures) of differences between individuals with and without psychotic experiences at age 24 years.

Table S3. Comparison for each covariate between individuals with and without negative symptoms at age 24 years in the observed, non-imputed data

|  |  |  | Negative symptoms at 24y | |  |
| --- | --- | --- | --- | --- | --- |
| Measure | |  | Yes | No | ***P*** |
| Psychotic experiences PRS | | N | 223 | 2158 | 0.12 |
|  |  | Mean | 0.14 | 0 |  |
|  |  | SD | 0.92 | 0.99 |  |
| Schizophrenia PRS | | N | 223 | 2158 | 0.18 |
|  |  | Mean | -0.01 | -0.09 |  |
|  |  | SD | 1.04 | 1 |  |
| Depression PRS | | N | 223 | 2158 | <0.001 |
|  |  | Mean | 0.1 | -0.1 |  |
|  |  | SD | 0.99 | 1.01 |  |
| Anxiety PRS | | N | 223 | 2158 | <0.001 |
|  |  | Mean | 0.18 | -0.05 |  |
|  |  | SD | 0.96 | 1.02 |  |
| CRP age 9 | | N | 169 | 1834 | 0.17 |
|  |  | Mean | 1.12 | 0.77 |  |
|  |  | SD | 3.67 | 2.69 |  |
| IL-6 age 9 | | N | 169 | 1831 | 0.02 |
|  |  | Mean | 1.56 | 1.26 |  |
|  |  | SD | 1.95 | 1.52 |  |
| BMI age 8 | | N | 231 | 2351 | 0.29 |
|  |  | Mean | 17.36 | 17.1 |  |
|  |  | SD | 2.82 | 2.32 |  |
| Sex | Male | N (%) | 119 (9.76) | 1100 (90.24) | 0.29 |
|  | Female | N (%) | 172 (8.66) | 1814 (91.34) |  |
| Mother smoked in pregnancy | No | N (%) | 210 (7.95) | 2433 (92.05) | <0.001 |
|  | Yes | N (%) | 75 (14.59) | 439 (85.41) |  |
| Father smoked in pregnancy | No | N (%) | 133 (8.41) | 1449 (91.59) | 0.07 |
|  | Yes | N (%) | 100 (10.55) | 848 (89.45) |  |
| Parental social class | Professional | N (%) | 11 (6.83) | 150 (93.17) | 0.35 |
|  | Managerial | N (%) | 81 (9.00) | 819 (91.00) |  |
|  | Skilled (non-manual) | N (%) | 67 (7.77) | 795 (92.23) |  |
|  | Skilled (manual) | N (%) | 69 (9.73) | 640 (90.27) |  |
|  | Partly skilled | N (%) | 37 (11.08) | 297 (88.92) |  |
|  | Unskilled | N (%) | 9 (12.00) | 66 (88.00) |  |
| Trauma 5-11 years | No | N (%) | 123 (6.50) | 1769 (93.50) | <0.001 |
|  | Yes | N (%) | 168 (12.78) | 1147 (87.22) |  |

**Note:** PRS, polygenic risk scores; SD, standard deviation; BMI, body mass index; *P*, *P* value for t-test (continuous measures) or chi-squared test (categorical measures) of differences between individuals with and without negative symptoms at age 24 years.

Table S4. Comparison for each covariate between individuals with and without depression at age 24 years in the observed, non-imputed data

|  |  |  | Depression at 24y | |  |
| --- | --- | --- | --- | --- | --- |
| Measure | |  | Yes | No | ***P*** |
| Psychotic experiences PRS | | N | 176 | 2285 | 0.99 |
|  |  | Mean | 0.04 | 0.01 |  |
|  |  | SD | 0.93 | 0.99 |  |
| Schizophrenia PRS | | N | 176 | 2285 | 0.04 |
|  |  | Mean | 0.04 | -0.09 |  |
|  |  | SD | 0.99 | 1.01 |  |
| Depression PRS | | N | 176 | 2285 | <0.001 |
|  |  | Mean | 0.13 | -0.11 |  |
|  |  | SD | 1.03 | 1.00 |  |
| Anxiety PRS | | N | 176 | 2285 | 0.01 |
|  |  | Mean | 0.11 | -0.05 |  |
|  |  | SD | 1.00 | 1.02 |  |
| CRP age 9 | | N | 143 | 1930 | 0.44 |
|  |  | Mean | 1.00 | 0.77 |  |
|  |  | SD | 2.69 | 2.74 |  |
| IL-6 age 9 | | N | 143 | 1927 | 0.21 |
|  |  | Mean | 1.46 | 1.27 |  |
|  |  | SD | 1.72 | 1.53 |  |
| BMI age 8 | | N | 194 | 2477 | 0.41 |
|  |  | Mean | 17.33 | 17.09 |  |
|  |  | SD | 2.62 | 2.34 |  |
| Sex | Male | N (%) | 61 (4.83) | 1202 (95.17) | <0.001 |
|  | Female | N (%) | 183 (8.90) | 1874 (91.10) |  |
| Mother smoked in pregnancy | No | N (%) | 176 (6.43) | 2561 (93.57) | <0.001 |
|  | Yes | N (%) | 64 (12.01) | 469 (87.99) |  |
| Father smoked in pregnancy | No | N (%) | 104 (6.41) | 1519 (93.59) | 0.01 |
|  | Yes | N (%) | 92 (9.23) | 905 (90.77) |  |
| Parental social class | Professional | N (%) | 12 (7.27) | 153 (92.73) | 0.96 |
|  | Managerial | N (%) | 68 (7.34) | 859 (92.66) |  |
|  | Skilled (non-manual) | N (%) | 59 (6.64) | 829 (93.36) |  |
|  | Skilled (manual) | N (%) | 57 (7.76) | 678 (92.24) |  |
|  | Partly skilled | N (%) | 27 (7.65) | 326 (92.35) |  |
|  | Unskilled | N (%) | 5 (6.25) | 75 (93.75) |  |
| Trauma 5-11 years | No | N (%) | 104 (5.34) | 1843 (94.66) | <0.001 |
|  | Yes | N (%) | 140 (10.18) | 1235 (89.82) |  |

**Note:** PRS, polygenic risk scores; SD, standard deviation; BMI, body mass index; *P*, *P* value for t-test (continuous measures) or chi-squared test (categorical measures) of differences between individuals with and without depression at age 24 years.

Table S5. Comparison for each covariate between individuals with and without anxiety disorder at age 24 years in the observed, non-imputed data

|  |  |  | Anxiety disorder at 24y | |  |
| --- | --- | --- | --- | --- | --- |
| Measure | |  | Yes | No | ***P*** |
| Psychotic experiences PRS | | N | 245 | 2203 | 0.36 |
|  |  | Mean | -0.01 | 0.01 |  |
|  |  | SD | 0.99 | 0.99 |  |
| Schizophrenia PRS | | N | 245 | 2203 | 0.09 |
|  |  | Mean | 0.01 | -0.09 |  |
|  |  | SD | 1.05 | 1.00 |  |
| Depression PRS | | N | 245 | 2203 | 0.02 |
|  |  | Mean | 0.01 | -0.11 |  |
|  |  | SD | 0.99 | 1.01 |  |
| Anxiety PRS | | N | 245 | 2203 | 0.02 |
|  |  | Mean | 0.07 | -0.05 |  |
|  |  | SD | 1.04 | 1.01 |  |
| CRP age 9 | | N | 188 | 1874 | 0.86 |
|  |  | Mean | 0.80 | 0.78 |  |
|  |  | SD | 2.06 | 2.78 |  |
| IL-6 age 9 | | N | 187 | 1872 | 0.11 |
|  |  | Mean | 1.46 | 1.26 |  |
|  |  | SD | 1.60 | 1.54 |  |
| BMI age 8 | | N | 263 | 2389 | 0.33 |
|  |  | Mean | 17.32 | 17.09 |  |
|  |  | SD | 2.60 | 2.33 |  |
| Sex | Male | N (%) | 87 (6.93) | 1168 (93.07) | <0.001 |
|  | Female | N (%) | 239 (11.7) | 1804 (88.3) |  |
| Mother smoked in pregnancy | No | N (%) | 243 (8.94) | 2475 (91.06) | <0.001 |
|  | Yes | N (%) | 77 (14.5) | 454 (85.5) |  |
| Father smoked in pregnancy | No | N (%) | 149 (9.24) | 1463 (90.76) | 0.08 |
|  | Yes | N (%) | 112 (11.35) | 875 (88.65) |  |
| Parental social class | Professional | N (%) | 15 (9.20) | 148 (90.80) | 0.71 |
|  | Managerial | N (%) | 96 (10.45) | 823 (89.55) |  |
|  | Skilled (non-manual) | N (%) | 83 (9.44) | 796 (90.56) |  |
|  | Skilled (manual) | N (%) | 69 (9.40) | 665 (90.60) |  |
|  | Partly skilled | N (%) | 30 (8.52) | 322 (91.48) |  |
|  | Unskilled | N (%) | 11 (13.92) | 68 (86.08) |  |
| Trauma 5-11 years | No | N (%) | 163 (8.42) | 1774 (91.58) | <0.001 |
|  | Yes | N (%) | 163 (11.96) | 1200 (88.04) |  |

**Note:** PRS, polygenic risk scores; SD, standard deviation; BMI, body mass index; *P*, *P* value for t-test (continuous measures) or chi-squared test (categorical measures) of differences between individuals with and without anxiety disorder at age 24 years.

Table S6. Details of all variables included in imputation models

| **Variables** | **Reasons for inclusion** | **Imputation method** | **% Missingness:  PRS analysis** | **% Missingness: trauma analysis** |
| --- | --- | --- | --- | --- |
| **Analysis variables** | | | | |
| Psychotic experiences PRS | Exposure | linear regression | 0.00 | 33.34 |
| Schizophrenia PRS | Exposure | linear regression | 0.00 | 33.34 |
| Depression PRS | Exposure | linear regression | 0.00 | 33.34 |
| Anxiety PRS | Exposure | linear regression | 0.00 | 33.34 |
| Trauma (age 5-11) | Exposure | logistic regression | 26.21 | 0.00 |
| IL-6 (age 9) | Mediator | predictive mean matching | 48.42 | 49.95 |
| CRP (age 9) | Mediator | predictive mean matching | 48.30 | 49.84 |
| Psychotic experiences (age 24) | Primary outcome | logistic regression | 65.22 | 62.51 |
| Negative symptoms (age 24) | Primary outcome | logistic regression | 65.78 | 63.14 |
| Depression (age 24) | Primary outcome | logistic regression | 64.59 | 61.82 |
| Anxiety disorder (age 24) | Primary outcome | logistic regression | 64.75 | 62.07 |
| Psychotic experiences (age 18) | Secondary outcome | logistic regression | 56.67 | 54.03 |
| Negative symptoms (age 16) | Secondary outcome | logistic regression | 65.78 | 63.14 |
| Depression (age 18) | Secondary outcome | logistic regression | 58.09 | 55.43 |
| Anxiety disorder (age 18) | Secondary outcome | logistic regression | 58.09 | 55.43 |
| Mother smoked in pregnancy | Covariate | logistic regression | 7.85 | 2.56 |
| Father smoked in pregnancy | Covariate | logistic regression | 28.38 | 22.85 |
| BMI (age 8) | Covariate | linear regression | 37.89 | 32.28 |
| Parental social class | Covariate | ordered logistic regression | 14.79 | 8.06 |
| Sex at birth | Covariate | logistic regression | 0.22 | 33.34 |
| **Auxiliary variables** | | | | |
| MFQ depression score at age 16 | Inform outcomes and missingness | predictive mean matching | 55.15 | 0.17 |
| CRP (age 16) | Inform mediator | predictive mean matching | 64.64 | 49.20 |
| IL-6 receptor genetic variant (rs2228145) | Inform mediator | ordered logistic regression | 0.10 | 65.51 |
| Maternal EPDS score | Inform outcomes and missingness | predictive mean matching | 12.69 | 33.40 |
| BMI (age 7) | Inform covariate | linear regression | 26.05 | 6.11 |
| Maternal family history of psychiatric illness | Inform outcomes and missingness | logistic regression | 9.99 | 21.87 |
| Paternal family history of psychiatric illness | Inform outcomes and missingness | logistic regression | 36.68 | 3.34 |
| Mother’s highest qualification | Inform outcomes and missingness | logistic regression | 10.08 | 30.77 |
| Home ownership | Inform outcomes and missingness | multinomial logistic regression | 10.73 | 3.32 |

**Note:** PRS, polygenic risk score; IL-6, interleukin-6; CRP, C-reactive protein; BMI, body mass index; MFQ, Mood and Feelings Questionnaire; EPDS, Edinburgh Postnatal Depression Scale. “% Missingness: PRS analysis” and “% Missingness: trauma analysis” refer to the variable missingness within the analysis sample when the PRSs were the exposures of interest and when trauma was the exposure of interest, respectively.

Table S7: Associations between psychiatric phenotype polygenic risk scores and psychopathology at age 24 years (n = 7859)

|  | **Psychotic experiences** | **Negative symptoms** | **Depression** | **Any anxiety disorder** |
| --- | --- | --- | --- | --- |
| **PRS** | **OR (95% CI) p value** | **OR (95% CI) p value** | **OR (95% CI) p value** | **OR (95% CI) p value** |
| **Psychotic experiences** | 1.08 (0.97 to 1.22) 0.171 | 1.15 (1.01 to 1.31) 0.040 | 1.05 (0.91 to 1.21) 0.498 | 0.96 (0.85 to 1.09) 0.531 |
| **Schizophrenia** | 1.22 (1.08 to 1.37) 0.001 | 1.11 (0.97 to 1.26) 0.115 | 1.15 (1.00 to 1.32) 0.055 | 1.1 (0.99 to 1.24) 0.083 |
| **Depression** | 1.18 (1.05 to 1.33) 0.007 | 1.34 (1.18 to 1.51) <0.001 | 1.41 (1.23 to 1.62) <0.001 | 1.22 (1.07 to 1.39) 0.003 |
| **Anxiety** | 1.14 (1.00 to 1.29) 0.053 | 1.30 (1.17 to 1.45) <0.001 | 1.26 (1.12 to 1.43) <0.001 | 1.19 (1.07 to 1.32) 0.002 |

**Note:** OR, odds ratio; 95% CI, confidence interval; PRS, polygenic risk score. All analyses were adjusted for the first 10 genetic principal components. Single nucleotide polymorphisms included in the PRS met a genome-wide association threshold of p ≤ 0.5.

Table S8: Associations between trauma (age 5-11) and psychopathology at age 24 years (n = 8700)

|  | **Psychotic experiences** | **Negative  symptoms** | **Depression** | **Any anxiety disorder** |
| --- | --- | --- | --- | --- |
| **Unadjusted  OR (95% CI)**  **p value** | 1.90 (1.50 to 2.41) <0.001 | 2.21 (1.73 to 2.81) <0.001 | 2.21 (1.72 to 2.83) <0.001 | 1.62 (1.31 to 2.00) <0.001 |
| **Adjusted  OR (95% CI)^a^**  **p value** | 1.87 (1.47 to 2.37) <0.001 | 2.16 (1.69 to 2.77) <0.001 | 2.31 (1.79 to 2.97) <0.001 | 1.69 (1.36 to 2.09) <0.001 |

**Note:** OR, odds ratio; 95% CI, confidence interval

^a^ adjusted for BMI at age 8, sex and parental social class

Table S9: Associations between psychiatric phenotype polygenic risk scores, trauma at age 5-11 years and inflammatory markers at age 9 years

|  | **IL-6** | **CRP** |  |
| --- | --- | --- | --- |
| **Exposures** | **Beta (95% CI)  p value** | **Beta (95% CI)  p value** | **N** |
| **Psychotic experiences  PRS** | 0.02 (-0.02 to 0.07)  0.325 | 0.05 (-0.02 to 0.13)  0.168 | 7859 |
| **Schizophrenia  PRS** | 0.02 (-0.03 to 0.07)  0.397 | -0.01 (-0.09 to 0.08)  0.836 | 7859 |
| **Depression  PRS** | 0.04 (-0.01 to 0.09)  0.101 | 0.04 (-0.04 to 0.12)  0.356 | 7859 |
| **Anxiety  PRS** | 0.004 (-0.05 to 0.06)  0.870 | 0.05 (-0.03 to 0.13)  0.194 | 7859 |
| **Trauma** | 0.05 (-0.04 to 0.14)  0.233 | 0.14 (-0.01 to 0.29)  0.060 | 8700 |

**Note:** IL-6, interleukin-6; CRP, C-reactive protein; 95% CI, confidence interval; PRS, polygenic risk score. All PRS analyses were adjusted for the first 10 genetic principal components. Single nucleotide polymorphisms included in the PRS met a genome-wide association threshold of p ≤ 0.5. Trauma analyses were adjusted for BMI at age 8, sex and parental social class

Table S10: Associations between inflammatory markers at age 9 years and psychopathology at age 24 years (n = 8700)

|  | **Psychotic experiences** | **Negative symptoms** | **Depression** | **Any anxiety disorder** |
| --- | --- | --- | --- | --- |
| **Inflammatory marker** | **OR (95% CI) p value** | **OR (95% CI) p value** | **OR (95% CI) p value** | **OR (95% CI) p value** |
| **IL-6** | 1.05 (0.98 to 1.14) 0.177 | 1.14 (1.05 to 1.23) 0.001 | 1.08 (0.97 to 1.20) 0.169 | 1.07 (0.99 to 1.17) 0.083 |
| **CRP** | 1.00 (0.96 to 1.05) 0.898 | 1.03 (1.00 to 1.07) 0.072 | 1.03 (0.99 to 1.08) 0.164 | 1.01 (0.97 to 1.06) 0.510 |

**Note:** OR, odds ratio; 95% CI, confidence interval; IL-6, interleukin-6; CRP, C-reactive protein. All analyses were adjusted for trauma (age 5-11 years), parental smoking, parental social class, sex and BMI (age 8)

Table S11: Mediation analysis results of the association between PRSs for psychiatric phenotypes and psychopathology at age 18 through IL-6 and CRP at age 9

| **PRS** | **Outcome** | **Total effect**  **OR (95% CI)** | **Direct effect**  **OR (95% CI)** | **Indirect effect**  **OR (95% CI)** |
| --- | --- | --- | --- | --- |
| **Mediation through IL-6** | | | | |
| Psychotic experiences | Psychotic experiences | 1.22  (1.01 to 1.48) | 1.22  (1.01 to 1.48) | 1.00  (0.99 to 1.01) |
| Schizophrenia | Psychotic experiences | 1.00  (0.82 to 1.22) | 1.00  (0.82 to 1.21) | 1.00  (0.99 to 1.01) |
| Depression | Negative symptoms | 1.21  (1.07 to 1.37) | 1.21  (1.07 to 1.37) | 1.00  (1.00 to 1.00) |
| Depression | Depression | 1.21  (1.03 to 1.41) | 1.21  (1.03 to 1.41) | 1.00  (0.99 to 1.01) |
| Depression | Any anxiety disorder | 1.14  (1.00 to 1.29) | 1.13  (1.00 to 1.29) | 1.00  (1.00 to 1.01) |
| Anxiety | Negative symptoms | 1.18  (1.05 to 1.33) | 1.18  (1.05 to 1.33) | 1.00  (1.00 to 1.00) |
| Anxiety | Depression | 1.21  (1.03 to 1.43) | 1.21  (1.03 to 1.43) | 1.00  (1.00 to 1.00) |
| Anxiety | Any anxiety disorder | 1.10  (0.99 to 1.23) | 1.10  (0.99 to 1.23) | 1.00  (1.00 to 1.00) |
| **Mediation through CRP** | | | | |
| Psychotic experiences | Psychotic experiences | 1.23  (1.02 to 1.49) | 1.23  (1.01 to 1.48) | 1.00  (1.00 to 1.01) |
| Schizophrenia | Psychotic experiences | 1.01  (0.82 to 1.23) | 1.01  (0.82 to 1.23) | 1.00  (0.99 to 1.00) |
| Depression | Negative symptoms | 1.21  (1.07 to 1.37) | 1.21  (1.07 to 1.37) | 1.00  (1.00 to 1.00) |
| Depression | Depression | 1.21  (1.03 to 1.41) | 1.21  (1.03 to 1.41) | 1.00  (1.00 to 1.00) |
| Depression | Any anxiety disorder | 1.14  (1 to 1.29) | 1.14  (1.00 to 1.29) | 1.00  (1.00 to 1.00) |
| Anxiety | Negative symptoms | 1.18  (1.05 to 1.33) | 1.18  (1.05 to 1.33) | 1.00  (1.00 to 1.00) |
| Anxiety | Depression | 1.21  (1.02 to 1.43) | 1.21  (1.02 to 1.43) | 1.00  (1.00 to 1.00) |
| Anxiety | Any anxiety disorder | 1.10  (0.99 to 1.23) | 1.10  (0.99 to 1.23) | 1.00  (1.00 to 1.00) |

**Note:** OR, odds ratio; 95% CI, confidence interval; PRS, polygenic risk score; IL-6, interleukin-6; CRP, C-reactive protein. All analyses were adjusted for trauma (age 5-11 years), parental smoking, parental social class, sex and BMI (age 8).

Table S12: Mediation analysis results of the association between trauma (age 5-10) and psychopathology at age 18 through IL-6 and CRP at age 9

| **Outcome** | **Total effect**  **OR (95% CI)** | **Direct effect**  **OR (95% CI)** | **Indirect effect**  **OR (95% CI)** |
| --- | --- | --- | --- |
| **Mediation through IL-6** | | | |
| Psychotic experiences | 2.12  (1.46 to 3.08) | 2.10  (1.44 to 3.06) | 1.01  (0.99 to 1.03) |
| Negative symptoms | 1.91  (1.52 to 2.39) | 1.91  (1.52 to 2.39) | 1.00  (0.99 to 1.01) |
| Depression | 1.78  (1.33 to 2.38) | 1.77  (1.33 to 2.38) | 1.00  (0.99 to 1.01) |
| Any anxiety disorder | 1.64  (1.31 to 2.04) | 1.63  (1.31 to 2.04) | 1.00  (1.00 to 1.01) |
| **Mediation through CRP** | | | |
| Psychotic experiences | 2.13  (1.47 to 3.08) | 2.11  (1.46 to 3.05) | 1.01  (1 to 1.02) |
| Negative symptoms | 1.91  (1.52 to 2.39) | 1.90  (1.52 to 2.39) | 1.00  (0.99 to 1.01) |
| Depression | 1.78  (1.33 to 2.38) | 1.77  (1.32 to 2.37) | 1.01  (1.00 to 1.02) |
| Any anxiety disorder | 1.64  (1.31 to 2.04) | 1.63  (1.31 to 2.04) | 1.00  (1.00 to 1.01) |

**Note:** OR, odds ratio; 95% CI, confidence interval; IL-6, interleukin-6; CRP, C-reactive protein. All analyses were adjusted for parental smoking, parental social class, sex and BMI (age 8) and polygenic scores for psychotic experiences, schizophrenia, depression and anxiety.

Table S13: Mediation analysis results of the association between PRSs for psychiatric phenotypes based on SNPs meeting different GWAS p-value thresholds (*P*_T_) and psychopathology at age 24 through IL-6

| **PRS** | **Outcome** | **Total effect**  **OR (95% CI)** | **Direct effect**  **OR (95% CI)** | **Indirect effect**  **OR (95% CI)** |
| --- | --- | --- | --- | --- |
| ***P*_T_ = 0.05** | | | | |
| Psychotic experiences | Psychotic experiences | 1.03  (0.91 to 1.16) | 1.03  (0.91 to 1.16) | 1.00  (1.00 to 1.00) |
| Schizophrenia | Psychotic experiences | 1.20  (1.06 to 1.36) | 1.20  (1.06 to 1.36) | 1.00  (1.00 to 1.00) |
| Depression | Negative symptoms | 1.26  (1.11 to 1.43) | 1.25  (1.10 to 1.42) | 1.00  (1.00 to 1.01) |
| Depression | Depression | 1.33  (1.15 to 1.54) | 1.33  (1.15 to 1.54) | 1.00  (1.00 to 1.01) |
| Depression | Any anxiety disorder | 1.15  (1.01 to 1.30) | 1.15  (1.01 to 1.30) | 1.00  (1.00 to 1.01) |
| Anxiety | Negative symptoms | 1.28  (1.15 to 1.43) | 1.28  (1.15 to 1.44) | 1.00  (0.99 to 1.00) |
| Anxiety | Depression | 1.16  (1.03 to 1.32) | 1.16  (1.03 to 1.32) | 1.00  (1.00 to 1.00) |
| Anxiety | Any anxiety disorder | 1.19  (1.07 to 1.32) | 1.19  (1.07 to 1.32) | 1.00  (0.99 to 1.00) |
| ***P*_T_ = 5e^-8^** | | | | |
| Psychotic experiences | Psychotic experiences | 0.95  (0.84 to 1.08) | 0.95  (0.84 to 1.08) | 1.00  (1.00 to 1.00) |
| Schizophrenia | Psychotic experiences | 1.13  (1.00 to 1.27) | 1.13  (1 to 1.27) | 1.00  (1.00 to 1.00) |
| Depression | Negative symptoms | 1.17  (1.03 to 1.34) | 1.17  (1.03 to 1.34) | 1.00  (1.00 to 1.01) |
| Depression | Depression | 1.11  (0.97 to 1.28) | 1.11  (0.97 to 1.28) | 1.00  (1.00 to 1.00) |
| Depression | Any anxiety disorder | 1.02  (0.91 to 1.15) | 1.02  (0.91 to 1.15) | 1.00  (1.00 to 1.01) |
| Anxiety | Negative symptoms | 0.99  (0.89 to 1.11) | 0.99  (0.89 to 1.10) | 1.01  (1.00 to 1.01) |
| Anxiety | Depression | 1.00  (0.88 to 1.13) | 0.99  (0.88 to 1.12) | 1.00  (1.00 to 1.01) |
| Anxiety | Any anxiety disorder | 0.99  (0.88 to 1.12) | 0.99  (0.88 to 1.12) | 1.00  (1.00 to 1.01) |

**Note:** OR, odds ratio; 95% CI, confidence interval; PRS, polygenic risk score; IL-6, interleukin-6. All analyses were adjusted for trauma (age 5-11 years), parental smoking, parental social class, sex and BMI (age 8).

Table S14: Mediation analysis results of the association between PRSs for psychiatric phenotypes based on SNPs meeting different GWAS p-value thresholds (*P*_T_) and psychopathology at age 24 through CRP

| **PRS** | **Outcome** | **Total effect**  **OR (95% CI)** | **Direct effect**  **OR (95% CI)** | **Indirect effect**  **OR (95% CI)** |
| --- | --- | --- | --- | --- |
| ***P*_T_ = 0.05** | | | | |
| Psychotic experiences | Psychotic experiences | 1.03  (0.91 to 1.16) | 1.03  (0.91 to 1.16) | 1.00  (1.00 to 1.00) |
| Schizophrenia | Psychotic experiences | 1.20  (1.06 to 1.36) | 1.20  (1.06 to 1.36) | 1.00  (1.00 to 1.00) |
| Depression | Negative symptoms | 1.26  (1.11 to 1.43) | 1.26  (1.11 to 1.43) | 1.00  (1.00 to 1.00) |
| Depression | Depression | 1.33  (1.15 to 1.54) | 1.33  (1.15 to 1.54) | 1.00  (1.00 to 1.00) |
| Depression | Any anxiety disorder | 1.15  (1.01 to 1.30) | 1.15  (1.01 to 1.30) | 1.00  (1.00 to 1.00) |
| Anxiety | Negative symptoms | 1.28  (1.15 to 1.43) | 1.28  (1.15 to 1.43) | 1.00  (1.00 to 1.00) |
| Anxiety | Depression | 1.16  (1.03 to 1.32) | 1.16  (1.03 to 1.32) | 1.00  (1.00 to 1.00) |
| Anxiety | Any anxiety disorder | 1.19  (1.07 to 1.32) | 1.19  (1.07 to 1.32) | 1.00  (1.00 to 1.00) |
| ***P*_T_ = 5e^-8^** | | | | |
| Psychotic experiences | Psychotic experiences | 0.95  (0.84 to 1.08) | 0.95  (0.84 to 1.08) | 1.00  (1.00 to 1.00) |
| Schizophrenia | Psychotic experiences | 1.13  (1.00 to 1.27) | 1.13  (1.00 to 1.27) | 1.00  (0.99 to 1.01) |
| Depression | Negative symptoms | 1.17  (1.03 to 1.34) | 1.18  (1.03 to 1.34) | 1.00  (0.99 to 1.00) |
| Depression | Depression | 1.11  (0.97 to 1.28) | 1.12  (0.97 to 1.28) | 1.00  (1.00 to 1.00) |
| Depression | Any anxiety disorder | 1.02  (0.91 to 1.15) | 1.02  (0.91 to 1.15) | 1.00  (1.00 to 1.00) |
| Anxiety | Negative symptoms | 1.00  (0.89 to 1.11) | 0.99  (0.89 to 1.11) | 1.00  (1.00 to 1.00) |
| Anxiety | Depression | 1.00  (0.88 to 1.13) | 1.00  (0.88 to 1.13) | 1.00  (1.00 to 1.00) |
| Anxiety | Any anxiety disorder | 0.99  (0.88 to 1.12) | 0.99  (0.88 to 1.12) | 1.00  (1.00 to 1.00) |

**Note:** OR, odds ratio; 95% CI, confidence interval; PRS, polygenic risk score; CRP, C-reactive protein. All analyses were adjusted for trauma (age 5-11 years), parental smoking, parental social class, sex and BMI (age 8).

Table S15: Complete case mediation analysis results of the association between PRSs for psychiatric phenotypes and psychopathology at age 24 through IL-6 and CRP at age 9

| **PRS** | **Outcome** | **N** | **Total effect**  **OR (95% CI)** | **Direct effect**  **OR (95% CI)** | **Indirect effect**  **OR (95% CI)** |
| --- | --- | --- | --- | --- | --- |
| **Mediation through IL-6** | | | | | |
| Psychotic experiences | Psychotic experiences | 1096 | 1.12  (0.89 to 1.42) | 1.12  (0.89 to 1.41) | 1.00  (1.00 to 1.01) |
| Schizophrenia | Psychotic experiences | 1096 | 1.27  (1.03 to 1.58) | 1.28  (1.03 to 1.58) | 1.00  (0.99 to 1.00) |
| Depression | Negative symptoms | 1078 | 1.41  (1.14 to 1.75) | 1.41  (1.14 to 1.74) | 1.00  (1.00 to 1.01) |
| Depression | Depression | 1112 | 1.68  (1.29 to 2.17) | 1.67  (1.29 to 2.17) | 1.00  (0.99 to 1.01) |
| Depression | Any anxiety disorder | 1106 | 1.14  (0.90 to 1.45) | 1.14  (0.90 to 1.45) | 1.00  (1.00 to 1.01) |
| Anxiety | Negative symptoms | 1078 | 1.31  (1.04 to 1.65) | 1.31  (1.04 to 1.64) | 1.00  (1.00 to 1.01) |
| Anxiety | Depression | 1112 | 1.29  (1.03 to 1.61) | 1.29  (1.03 to 1.61) | 1.00  (1.00 to 1.01) |
| Anxiety | Any anxiety disorder | 1106 | 1.21  (0.98 to 1.50) | 1.21  (0.98 to 1.49) | 1.00  (1.00 to 1.01) |
| **Mediation through CRP** | | | | | |
| Psychotic experiences | Psychotic experiences | 1098 | 1.12  (0.88 to 1.43) | 1.13  (0.88 to 1.44) | 1.00  (0.99 to 1.01) |
| Schizophrenia | Psychotic experiences | 1098 | 1.27  (1.03 to 1.57) | 1.27  (1.03 to 1.57) | 1.00  (0.99 to 1.01) |
| Depression | Negative symptoms | 1080 | 1.41  (1.13 to 1.76) | 1.41  (1.13 to 1.76) | 1.00  (0.99 to 1.01) |
| Depression | Depression | 1114 | 1.67  (1.28 to 2.19) | 1.67  (1.28 to 2.19) | 1.00  (0.99 to 1.01) |
| Depression | Any anxiety disorder | 1108 | 1.16  (0.92 to 1.46) | 1.16  (0.93 to 1.46) | 1.00  (0.99 to 1.00) |
| Anxiety | Negative symptoms | 1080 | 1.30  (1.04 to 1.63) | 1.31  (1.04 to 1.63) | 1.00  (0.99 to 1.01) |
| Anxiety | Depression | 1114 | 1.29  (1.03 to 1.61) | 1.29  (1.04 to 1.61) | 1.00  (0.99 to 1.01) |
| Anxiety | Any anxiety disorder | 1108 | 1.22  (0.99 to 1.51) | 1.23  (0.99 to 1.51) | 1.00  (0.99 to 1.01) |

**Note:** OR, odds ratio; 95% CI, confidence interval; PRS, polygenic risk score; IL-6, interleukin-6; CRP, C-reactive protein; N, number of participants with data on exposure, outcome, mediator, and covariates. All analyses were adjusted for trauma (age 5-11 years), parental smoking, parental social class, sex and BMI (age 8)

Table S16: Complete case mediation analysis results of the association between trauma (age 5-10) and psychopathology at age 24 through IL-6 and CRP at age 9

| **Outcome** | **N** | **Total effect**  **OR (95% CI)** | **Direct effect**  **OR (95% CI)** | **Indirect effect**  **OR (95% CI)** |
| --- | --- | --- | --- | --- |
| **Mediation through IL-6** | | | | |
| Psychotic experiences | 1299 | 1.53  (0.96 to 2.45) | 1.53  (0.96 to 2.44) | 1.00  (0.99 to 1.02) |
| Negative symptoms | 1276 | 1.93  (1.22 to 3.07) | 1.92  (1.21 to 3.05) | 1.00  (0.99 to 1.02) |
| Depression | 1320 | 2.48  (1.47 to 4.20) | 2.48  (1.46 to 4.19) | 1.00  (0.99 to 1.01) |
| Any anxiety disorder | 1310 | 1.57  (1.01 to 2.45) | 1.57  (1.00 to 2.45) | 1.00  (0.99 to 1.02) |
| **Mediation through CRP** | | | | |
| Psychotic experiences | 1302 | 1.57  (0.99 to 2.49) | 1.58  (0.99 to 2.50) | 1.00  (0.98 to 1.01) |
| Negative symptoms | 1279 | 1.98  (1.25 to 3.16) | 1.99  (1.25 to 3.16) | 1.00  (0.98 to 1.01) |
| Depression | 1323 | 2.54  (1.53 to 4.21) | 2.54  (1.53 to 4.22) | 1.00  (0.98 to 1.02) |
| Any anxiety disorder | 1313 | 1.56  (0.98 to 2.48) | 1.56  (0.98 to 2.49) | 1.00  (0.98 to 1.01) |

**Note:** OR, odds ratio; 95% CI, confidence interval; IL-6, interleukin-6; CRP, C-reactive protein; N, number of participants with data on exposure, outcome, mediator, and covariates. All analyses were adjusted for parental smoking, parental social class, sex and BMI (age 8) and polygenic scores for psychotic experiences, schizophrenia, depression and anxiety. Standard errors for indirect effects were calculated using bootstrapping with 500 replications.

Table S17: Complete case mediation analysis results of the association between PRSs for psychiatric phenotypes and psychopathology at age 24 through IL-6 and CRP at age 9 following removal of individuals with CRP levels > 10mg/L or who reported an infection at the time of blood collection or in the preceding week

| **PRS** | **Outcome** | **N** | **Total effect**  **OR (95% CI)** | **Direct effect**  **OR (95% CI)** | **Indirect effect**  **OR (95% CI)** |
| --- | --- | --- | --- | --- | --- |
| **Mediation through IL-6** | | | | | |
| Psychotic experiences | Psychotic experiences | 988 | 1.13  (0.88 to 1.45) | 1.13  (0.88 to 1.45) | 1.00  (1.00 to 1.00) |
| Schizophrenia | Psychotic experiences | 988 | 1.26  (1.02 to 1.56) | 1.26  (1.02 to 1.56) | 1.00  (1.00 to 1.00) |
| Depression | Negative symptoms | 972 | 1.37  (1.07 to 1.74) | 1.36  (1.07 to 1.73) | 1.00  (1.00 to 1.01) |
| Depression | Depression | 1004 | 1.57  (1.19 to 2.08) | 1.57  (1.18 to 2.08) | 1.00  (1.00 to 1.01) |
| Depression | Any anxiety disorder | 1000 | 1.09  (0.85 to 1.39) | 1.08  (0.84 to 1.39) | 1.00  (1.00 to 1.01) |
| Anxiety | Negative symptoms | 972 | 1.29  (1.00 to 1.65) | 1.29  (1.00 to 1.65) | 1.00  (0.99 to 1.01) |
| Anxiety | Depression | 1004 | 1.23  (0.98 to 1.55) | 1.23  (0.98 to 1.55) | 1.00  (1.00 to 1.01) |
| Anxiety | Any anxiety disorder | 1000 | 1.22  (0.96 to 1.55) | 1.22  (0.96 to 1.55) | 1.00  (0.99 to 1.01) |
| **Mediation through CRP** | | | | | |
| Psychotic experiences | Psychotic experiences | 990 | 1.13  (0.88 to 1.45) | 1.13  (0.88 to 1.45) | 1.00  (0.99 to 1.01) |
| Schizophrenia | Psychotic experiences | 990 | 1.26  (1.02 to 1.56) | 1.27  (1.03 to 1.57) | 1.00  (0.99 to 1.01) |
| Depression | Negative symptoms | 974 | 1.36  (1.08 to 1.72) | 1.36  (1.08 to 1.71) | 1.00  (0.99 to 1.01) |
| Depression | Depression | 1006 | 1.57  (1.18 to 2.08) | 1.57  (1.18 to 2.08) | 1.00  (0.99 to 1.01) |
| Depression | Any anxiety disorder | 1002 | 1.10  (0.86 to 1.41) | 1.10  (0.86 to 1.41) | 1.00  (0.99 to 1.01) |
| Anxiety | Negative symptoms | 974 | 1.28  (1.00 to 1.62) | 1.27  (1.00 to 1.62) | 1.00  (0.99 to 1.02) |
| Anxiety | Depression | 1006 | 1.23  (0.98 to 1.54) | 1.22  (0.97 to 1.54) | 1.00  (0.99 to 1.02) |
| Anxiety | Any anxiety disorder | 1002 | 1.23  (0.97 to 1.56) | 1.23  (0.97 to 1.55) | 1.00  (0.99 to 1.01) |

**Note:** OR, odds ratio; 95% CI, confidence interval; PRS, polygenic risk score; IL-6, interleukin-6; CRP, C-reactive protein; N, number of participants with data on exposure, outcome, mediator, and covariates. All analyses were adjusted for trauma (age 5-11 years), parental smoking, parental social class, sex and BMI (age 8). Standard errors for indirect effects were calculated using bootstrapping with 500 replications.

Table S18: Complete case mediation analysis results of the association between trauma (age 5-10) and psychopathology at age 24 through IL-6 and CRP at age 9 following removal of individuals with CRP levels > 10mg/L or who reported an infection at the time of blood collection or in the preceding week

| **Outcome** | **N** | **Total effect**  **OR (95% CI)** | **Direct effect**  **OR (95% CI)** | **Indirect effect**  **OR (95% CI)** |
| --- | --- | --- | --- | --- |
| **Mediation through IL-6** | | | | |
| Psychotic experiences | 1168 | 1.71  (1.05 to 2.78) | 1.71  (1.05 to 2.77) | 1.00  (0.99 to 1.02) |
| Negative symptoms | 1149 | 1.82  (1.09 to 3.03) | 1.81  (1.08 to 3.01) | 1.01  (0.99 to 1.03) |
| Depression | 1189 | 2.49  (1.41 to 4.40) | 2.48  (1.41 to 4.38) | 1.00  (0.99 to 1.02) |
| Any anxiety disorder | 1181 | 1.67  (1.01 to 2.76) | 1.66  (1.01 to 2.74) | 1.01  (0.99 to 1.03) |
| **Mediation through CRP** | | | | |
| Psychotic experiences | 1171 | 1.67  (1.00 to 2.8) | 1.66  (0.99 to 2.79) | 1.01  (0.98 to 1.03) |
| Negative symptoms | 1152 | 1.82  (1.14 to 2.89) | 1.81  (1.13 to 2.88) | 1.01  (0.99 to 1.03) |
| Depression | 1192 | 2.49  (1.37 to 4.52) | 2.48  (1.37 to 4.51) | 1.00  (0.99 to 1.02) |
| Any anxiety disorder | 1184 | 1.58  (0.97 to 2.57) | 1.57  (0.96 to 2.56) | 1.01  (0.99 to 1.03) |

**Note:** OR, odds ratio; 95% CI, confidence interval; IL-6, interleukin-6; CRP, C-reactive protein; N, number of participants with data on exposure, outcome, mediator, and covariates. All analyses were adjusted for parental smoking, parental social class, sex and BMI (age 8) and polygenic scores for psychotic experiences, schizophrenia, depression and anxiety. Standard errors for indirect effects were calculated using bootstrapping with 500 replications.
